# Supplementary material for: Optical Coherence Tomography Angiography in Diabetic Patients: A Systematic Review
Source: Biomedicines. 2021 Dec 31;10(1):88. doi: 10.3390/biomedicines10010088 (PMC8773551; doi:10.3390/biomedicines10010088)
Supplement: Supplementary file 1 [file biomedicines-10-00088-s001.zip › biomedicines-1507235-supplementary.pdf]

SUPPLEMENTARY FILE S1

Morphology of the retinal and choroidal blood vessels

The neural retina is a nervous tissue comprised of nine layers described from the inner to the outer retina: inner limiting membrane (ILM), retinal nerve fiber layer (RNFL), ganglion cell layer (GCL), inner plexiform layer (IPL), inner nuclear layer (INL), outer plexiform layer (OPL), outer nuclear layer (ONL), external limiting membrane (ELM), and photoreceptor segments (PS). The interdigitations of the retinal pigment epithelium (RPE) surround the outer segment photoreceptors [1].

The high metabolic activity of the retina requires a large blood supply achieved through two different vascular systems. The inner two-thirds of the retina until the INL depends on blood supply from the central retinal artery. The blood supply for the outer retina depends on choroid vascularization [2]. The central retinal artery, a terminal branch of the ophthalmic artery, enters through the optic nerve and divides and forms different plexuses covering the entire retina, excluding the central foveal avascular zone (FAZ) and the most peripheral 1-1.5 mm [3–6]. The number of retinal plexuses varies from 1 to 4 depending on the eccentricity [7]. Close to the optic nerve head, the radial peripapillary capillary network (RPCN), the superficial vascular or capillary plexus (SVP/SCP), the intermediate capillary plexus (ICP), and the deep capillary plexus (DCP) exist [8]. The rest of the central retina is formed by the SCP, ICP, and DCP [8] (Figure 1).

The FAZ is the capillary-free region around the fovea [4]. The FAZ is delimited by a vascular ring at the ICP level resulting from the convergence of the three main vascular plexuses (SCP, ICP, and DCP). The blood supply to the fovea arrives from the underlying choroidal circulation [9].

The RPCN is a vascular plexus mainly located in the RNFL around the optic nerve and is composed of a simple layer of capillaries interconnected with each other and arranged parallel to the fibers [8,10]. The SCP comprises a three-dimensional vessel network at the GCL level formed by larger arteries, arterioles, veins, and venules [11]. This plexus presents a capillary-free zone in the central retina [10,12] and branches mainly into straight vessels. The ICP forms a simple vascular layer with tortuous vessels between the INL and IPL and disappears progressively towards the retinal periphery connecting the SCP and the DCP. Finally, the DCP is a single layer of vessels located in the outermost part of the INL formed by closed capillary loops and spider-like structures [8,12].

Vascular diseases, including DM, or other retinal diseases, could change the morphology and size of the FAZ in the different plexuses. Imaging techniques such as OCTA can detect and analyse vascular alterations such as MA, capillary dropout, slow blood flow areas, IRMA, or NV, among others [13–15].

Otherwise, this technique also eases the study of the choroid vessels responsible for nourishing the outer retina. The choroid is a complex structure mainly formed by vessels and connective tissue that extends from the limits of the optic nerve. Bruch's

membrane, the choriocapillaris (CC), Haller's and Sattler's layers, and the suprachoroidal space are included [16].

1. Kolb, H. Simple Anatomy of the Retina. In *Webvision: The Organization of the Retina and Visual System [Internet].*; Kolb, H., Fernandez, E., Nelson, R., Eds.; Salt Lake City (UT), University of Utah Health Sciences Center.
2. Bill, A. Circulation in the Eye. In *Handbook in Physiology. Microcirculation*; EM, R., Ed.; 1984; pp. 1001–1034.
3. Hogan, MJ; Alvarado, JA; Wedell, J. Retina, Histology of the Human Eye. In; Saunders, Ed.; 1971; pp. 508–519.
4. Provis, J.M. Development of the primate retinal vasculature. *Prog. Retin. Eye Res.* **2001**, *20*, 799–821, doi:10.1016/s1350-9462(01)00012-x.
5. Snodderly, D.M.; Weinhaus, R.S.; Choi, J.C. Neural-vascular relationships in central retina of macaque monkeys (Macaca fascicularis). *J. Neurosci.* **1992**, *12*, 1169–1193, doi:10.1523/JNEUROSCI.12-04-01169.1992.
6. Hayreh, S. Phisiological anatomy of the retinal vasculature. In *Immunology, inflammation and diseases of the Eye*; Dartt, D; D´Amore, P; Niederkorn, J., Ed.; Elsevier, 2010; pp. 207–212.
7. Campbell, J.P.; Zhang, M.; Hwang, T.S.; Bailey, S.T.; Wilson, D.J.; Jia, Y.; Huang, D. Detailed Vascular Anatomy of the Human Retina by Projection-Resolved Optical Coherence Tomography Angiography. *Sci. Rep.* **2017**, *7*, 1–11, doi:10.1038/srep42201.
8. Cuenca, N.; Ortuño-Lizarán, I.; Sánchez-Sáez, X.; Kutsyr, O.; Albertos-Arranz, H.; Fernández-Sánchez, L.; Martínez-Gil, N.; Noailles, A.; López-Garrido, J.A.; López-Gálvez, M.; et al. Interpretation of OCT and OCTA images from a histological approach: Clinical and experimental implications. *Prog. Retin. Eye Res.* **2020**, *77*, 100828, doi:10.1016/j.preteyeres.2019.100828.
9. Delaey, C.; Van De Voorde, J. Regulatory mechanisms in the retinal and choroidal circulation. *Ophthalmic Res.* **2000**, *32*, 249–256, doi:10.1159/000055622.
10. Chandrasekera, E.; An, D.; McAllister, I.L.; Yu, D.-Y.; Balaratnasingam, C. Three-Dimensional Microscopy Demonstrates Series and Parallel Organization of Human Peripapillary Capillary Plexuses. *Invest. Ophthalmol. Vis. Sci.* **2018**, *59*, 4327–4344, doi:10.1167/iovs.18-24105.
11. Spaide, R.F.; Fujimoto, J.G.; Waheed, N.K.; Sadda, S.R.; Staurenghi, G. Optical coherence tomography angiography. *Prog. Retin. Eye Res.* **2018**, *64*, 1–55, doi:10.1016/j.preteyeres.2017.11.003.
12. Balaratnasingam, C.; An, D.; Sakurada, Y.; Lee, C.S.; Lee, A.Y.; McAllister, I.L.; Freund, K.B.; Sarunic, M.; Yu, D.-Y. Comparisons Between Histology and Optical Coherence Tomography Angiography of the Periarterial Capillary-Free Zone. *Am. J. Ophthalmol.* **2018**, *189*, 55–64, doi:10.1016/j.ajo.2018.02.007.
13. Tam, J.; Dhamdhere, K.P.; Tiruveedhula, P.; Lujan, B.J.; Johnson, R.N.; Bearse, M.A.J.; Adams, A.J.; Roorda, A. Subclinical capillary changes in non-proliferative diabetic retinopathy. *Optom. Vis. Sci.* **2012**, *89*, E692-703, doi:10.1097/OPX.0b013e3182548b07.
14. Matsunaga, D.R.; Yi, J.J.; De Koo, L.O.; Ameri, H.; Puliafito, C.A.; Kashani, A.H.

Optical Coherence Tomography Angiography of Diabetic Retinopathy in Human Subjects. *Ophthalmic Surg Lasers Imaging Retin.* **2015**, *46*, 796–805, doi:10.3928/23258160-20150909-03.

15. Pappuru, R.K.R.; Ribeiro, L.; Lobo, C.; Alves, D.; Cunha-Vaz, J. Microaneurysm turnover is a predictor of diabetic retinopathy progression. *Br. J. Ophthalmol.* **2019**, *103*, 222–226, doi:10.1136/bjophthalmol-2018-311887.
16. Borrelli, E.; Sarraf, D.; Freund, K.B.; Sadda, S.R. OCT angiography and evaluation of the choroid and choroidal vascular disorders. *Prog. Retin. Eye Res.* **2018**, *67*, 30–55, doi:10.1016/j.preteyeres.2018.07.002.
